# ARE POSTS ABOUT TRIGGER FINGER ON THE SOCIAL NETWORK *INSTAGRAM* OF ADEQUATE QUALITY AND RELIABILITY?

**DOI:** 10.1590/1413-785220263405e304605

**Published:** 2026-08-10

**Authors:** Luiz Felipi Alves Bizareli, Heloisa Yumi Fujiya Sungaila, João Carlos Belloti, Marcel Jun Sugawara Tamaoki, João Baptista Gomes dos Santos, Luis Renato Nakachima, Vinicius Ynoe de Moraes

**Affiliations:** 1 Escola Paulista de Medicina, Departamento de Ortopedia e Traumatologia, Universidade Federal de Sao Paulo (UNIFESP), Sao Paulo, SP, Brazil.

**Keywords:** Social Media, Messaging, Social Media, Trigger Finger Disorder, Stenosing Tenosynovitis, Internet, Mídias Sociais, Meios de Comunicação Sociais, Dedo em Gatilho, Tenossinovite Estenosante, Internet

## Abstract

**Introduction:**

Trigger finger (stenosing tenosynovitis of the flexors) is a common hand condition, causing pain and functional limitation. With the increasing use of social media as a source of information, especially Instagram, it becomes necessary to evaluate the quality and reliability of content related to the topic made available to the public.

**Objective:**

To evaluate the quality and reliability of information about trigger finger published on Instagram, using the validated instruments modified DISCERN and Global Quality Score (GQS).

**Methods:**

Observational, cross-sectional study based on the analysis of the hashtags #triggerfinger and #stenosingtenosynovitis. 118 eligible posts were analyzed. Two independent evaluators (orthopedic residents in hand surgery) classified the content.

**Results:**

There was excellent inter-rater agreement, and a positive correlation was observed between longer content duration and better quality. There was a significant difference between professionals: hand surgeons presented higher quality scores.

**Conclusion:**

The quality of the information is very low to moderate. Content produced by hand surgeons presents greater technical rigor, and the Reels format offers better informational quality. ***Level of evidence V; Descriptive observational study.***

## RESUMO

**Introdução:**

O dedo em gatilho (tenossinovite estenosante dos flexores) é uma condição comum da mão, causando dor e limitação funcional. Com o crescente uso de redes sociais como fonte de informação, especialmente o Instagram, torna-se necessário avaliar a qualidade e confiabilidade dos conteúdos relacionados ao tema disponibilizados ao público.

**Objetivo:**

Avaliar a qualidade e a confiabilidade das informações sobre dedo em gatilho publicadas no Instagram, utilizando os instrumentos validados DISCERN modificado e Global Quality Score (GQS).

**Métodos:**

Estudo observacional, transversal, baseado na análise com as hashtags #dedoemgatilho e #tenossinoviteestenosante. Foram analisados 118 posts elegíveis. Dois avaliadores independentes (ortopedistas residentes em cirurgia da mão) classificaram os conteúdos.

**Resultados:**

Houve excelente concordância interavaliadores e observou-se correlação positiva entre maior duração do conteúdo e melhor qualidade. Houve diferença significativa entre profissionais: cirurgiões de mão apresentaram maiores escores de qualidade.

**Conclusão:**

A qualidade das informações é muito baixa a moderada. Conteúdos produzidos por cirurgiões de mão apresentam maior rigor técnico, e o formato Reels apresenta melhor qualidade informacional. ***Nível de evidência V; Estudo observacional descritivo.***

## INTRODUCTION

Trigger finger (stenosing tenosynovitis of the flexor tendon at the A1 pulley) was a term first proposed by Notta in 1850.^1^ This condition is one of the most common causes of pain and disability in the hand, leading to varying degrees of limitation of daily activities.^2^ It presents with hand pain, edema, discomfort, and disability, with a "triggering" sensation.

Trigger finger is characterized by blocked gliding movements of the flexor tendon during finger flexion and extension at the topography of the A1 pulley.^3^ These pathological changes lead to an increase in the size of the flexor tendon and its tendon sheath, resulting in the inability to flex or extend the finger without discomfort.^4^

It has an annual incidence in the general population of 28 per 100,000 people.^5^ Among adults, women in the 5th and 6th decades of life are the most affected by trigger finger.^4^ In addition, trigger finger may be associated with other conditions, including carpal tunnel syndrome, gout, rheumatoid arthritis, De Quervain's disease, and diabetes *mellitus*.^4^

In general, the diagnosis is clinical, with a classic presentation of "clicking" and locking of the finger. However, ultrasonography or magnetic resonance imaging (MRI) may aid in the differential diagnosis with other conditions.^3^

Currently, several treatment options are available for trigger finger, including noninvasive and surgical procedures.^2^ Conservative treatment is initially chosen, with the use of splints, physiotherapy, nonsteroidal anti-inflammatory drugs (NSAIDs), and corticosteroid injection. Despite the good results of steroid treatment, many patients with trigger finger still require surgical therapy, with open or percutaneous release of the A1 pulley.^3^

Although the diagnosis of trigger finger is clinical and relatively simple, many patients turn to the internet to better understand this condition, its symptoms, and forms of treatment. Studies show that interest in online information about trigger finger has increased in recent years, reflecting the growing search for self-diagnosis and understanding of the disease by patients.^6^

Access to medical information through the internet has become a common practice among patients seeking to understand symptoms, diagnoses, and treatment options before consulting a health professional. However, the quality and accuracy of this information vary considerably, which can lead to misinterpretations and inappropriate clinical decisions.

An analysis of medical images related to common hand conditions revealed that 55% of the images about trigger finger contained errors, most of them originating from the websites of private and academic medical institutions.^7^

Furthermore, the way information is presented significantly influences patient understanding. A randomized clinical trial demonstrated that educational videos provide better understanding and greater satisfaction among patients with trigger finger compared with printed leaflets.^8^

In view of this scenario, it becomes essential to critically evaluate the quality, reliability, and accuracy of the information about trigger finger available on the internet, especially Instagram, one of the most common social media platforms, which presents itself as a fast and widely disseminated tool today, with Brazil being the third country with the largest number of users, totaling 134 million.^9^

However, the quality of the information provided by this platform appears questionable, as already evidenced by previous studies conducted on other topics, such as prostate cancer,^10^ oral cavity cancer,^11^ and hypothyroidism.^12^

By identifying the reliability and quality of this information, it becomes possible to confirm the accuracy of the data conveyed to patients, as well as to prevent misunderstandings and to preemptively address any doubts that may arise.

The hypothesis of this study is that the content available on Instagram about trigger finger/flexor stenosing tenosynovitis is of low quality and reliability.

**Figure 1:**
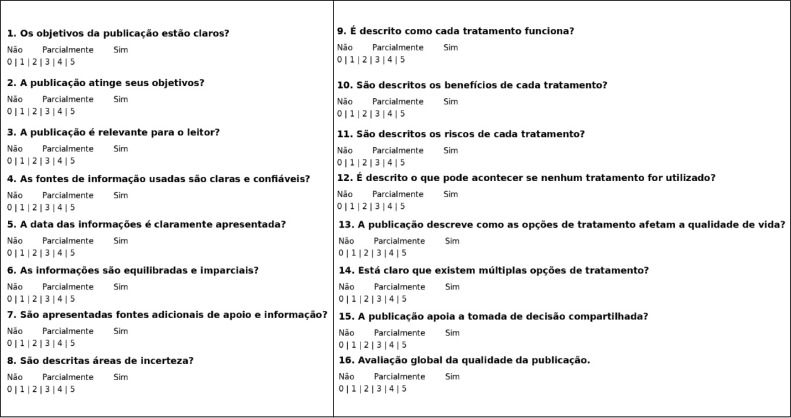
DISCERN questionnaire translated into Portuguese.

**Figure 2:**
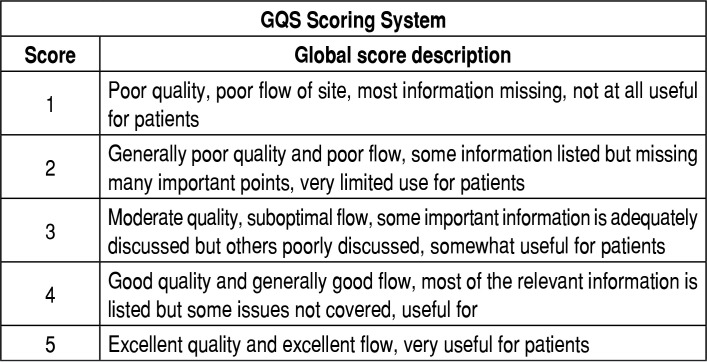
GQS Scoring System.

Therefore, this study proposed a systematic analysis of Instagram posts with the hashtags #dedoemgatilho and #tenossinoviteestenosante, using the modified DISCERN score (Figure 1), translated into Portuguese,^13^ and the Global Quality Score.^14^ (Figure 2)

## OBJECTIVE

To evaluate the quality of posts about trigger finger on the social media platform Instagram, using two different objective scores: modified DISCERN and GQS, in addition to inter-observer reliability.

## MATERIALS AND METHODS

Ethical approval was not required, as this study does not involve any human or animal participants.

A scientifically based, exploratory, cross-sectional study that analyzed, on a single day, the 118 most relevant posts with the hashtags #dedoemgatilho (68 posts) and #tenossinoviteestenosante (50 posts), from a newly created Instagram account, configured as Brazilian and belonging to a fictitious female user, approximately 50 years of age.

Content that was not in Portuguese, was not publicly open, or was unrelated to trigger finger was excluded by two of the evaluators (LFAB and HYS); in case of disagreement, a third evaluator (VYM) would decide on inclusion. The included posts were independently evaluated by two different evaluators, second-year orthopedic residents in hand surgery.

The scores used to evaluate the content were the modified DISCERN and the GQS. DISCERN^15^ was jointly developed by the University of Oxford and the British Library team (adapted from DISCERN, a tool created to evaluate written health content); it assesses the reliability, quality, and relevance of publications, and was translated into Portuguese by Logullo P. et al.^13^

The scoring system consists of 16 questions: the first 8 assess the reliability of the publication, the next 7 questions assess the quality of the details about the treatment of the condition, and the 16th question assesses the overall quality of the publication. Each question is scaled from 1 to 5 points; a score of 1 indicates a lack of information, a score between 2 and 3 indicates that some information is present, and a score of 5 indicated all information available. The total score ranges from 15 to 80, and the score results are rated as excellent (63–80), good (51–62), moderate (39–50), poor (28–38), and very poor (16–27).

The Global Quality Score (GQS) (14) assesses the quality of the content on a scale from 1 to 5, with 1 for content with low quality of information, missing information, and little usefulness for patients, and 5 points for content with excellent quality of information, being relevant and useful for the patient.

## RESULTS

We set a significance level of 0.05 (5%). Thus, the results are statistically significant when they are lower than the adopted significance level.

Nonparametric statistical tests were used, as we tested the normality of the quantitative primary outcome variables using the Shapiro-Wilk test (N≥100) and concluded that a normal distribution is not assured.

We performed a descriptive analysis of the two scores for each evaluator, but considering the total sample. A total sample size of N = 118 publications was obtained, with results according to Table 1.

**Table 1:** Complete Descriptive Statistics of DISCERN and GQS in the Total Sample.

			Mean	Median	Standard Deviation	Q1	Q3	N	CI
DISCERN	Evaluator 1	Trigger finger	24.02	24.0	5.56	21.0	26.8	118	1.00
	Evaluator 2	Trigger finger	22.25	21.0	5.13	19.0	25.0	118	0.93
GQS	Evaluator 1	Trigger finger	2.09	2.0	0.84	2.0	2.0	118	0.15
	Evaluator 2	Trigger finger	1.99	2.0	0.85	1.0	2.0	118	0.15

Analyzing, for example, DISCERN Evaluator 1 (DISCERN 1), we obtained a mean of 24.02 ± 1.00. Considering the same Evaluator 1, but now for GQS (GQS 1), the mean was 2.09 ± 0.15. For the DISCERN score by Evaluator 2 (DISCERN 2), we obtained a mean of 22.25 ± 0.93. Considering the same Evaluator 2, but now for GQS (GQS 2), the mean was 1.99 ± 0.15.

In addition, the agreement between the two evaluators was assessed for the results of the DISCERN and GQS questionnaires. Table 2 demonstrates that there is statistically significant agreement between the evaluators, both for DISCERN and for GQS. The ICC (Intraclass Correlation Coefficient) values are quite high.

**Table 2:** Agreement between Evaluators.

		ICC	p-value
Trigger finger	DISCERN	0.890	<0.001
	GQS	0.937	<0.001

The posts show a statistically significant correlation of duration with both DISCERN and GQS. We note that all correlations are positive, demonstrating that the longer the duration, the higher the DISCERN or GQS result.

We categorized the number of views as Low (fewer than 3,000) and High (more than 3,000) in order to analyze the relationship with the Professional. Table 3 shows the joint distribution of the variables in absolute values and their percentages across all combinations of the levels of these two variables. To verify whether or not there is an association (difference), we should examine the percentage values, by row, comparing the columns.

**Table 3:** Relationship of Views with the Professional.

N		Low		High		p-value
%	N	%				
Trigger finger	Hand surgeon	6	33.3%	10	27.8%	0.673
	Physiotherapist	5	27.8%	15	41.7%	0.319
	Physician	3	16.7%	3	8.3%	0.358
	Orthopedist	2	11.1%	2	5.6%	0.462
	Others	0	0.0%	2	5.6%	0.308
	Occupational therapist	2	11.1%	4	11.1%	1.000

No statistical correlation was identified between views and the professional; that is, the results are considered statistically independent. It is worth noting that we did not consider the professional groups that had only 2 cases.

The data in Table 4 provide information to compare the relationship between the number of views and the scores; the table demonstrates that there is no statistical difference between Views (Low/High) and the quality results of DISCERN and GQS.

**Table 4:** Relationship of Views (high vs. low) with the scores.

			Mean	Median	Standard Deviation	Q1	Q3	N	CI	p-value
DISCERN 1	Trigger finger	Low	25.56	25.5	5.73	23.0	28.8	18	2.65	0.769
		High	25.36	24.5	6.35	22.0	29.0	36	2.07	
DISCERN 2	Trigger finger	Low	23.61	21.0	5.63	20.0	27.5	18	2.60	0.733
		High	23.83	22.5	5.95	19.8	28.0	36	1.94	
GQS 1	Trigger finger	Low	2.44	2.0	0.86	2.0	3.0	18	0.40	0.797
		High	2.42	2.0	0.91	2.0	3.0	36	0.30	
GQS 2	Trigger finger	Low	2.33	2.0	0.84	2.0	3.0	18	0.39	0.968
		High	2.33	2.0	0.93	2.0	3.0	36	0.30	

The 5 professional groups were analyzed, with the data shown in Tables 5 and 6, in which it was possible to demonstrate that there is a statistically significant difference among them in the DISCERN 1 and 2 evaluations, as well as GQS 2 (p-values in Table 5).

**Table 5:** Comparison of the Professional for DISCERN and GQS.

		Mean	Median	Standard Deviation	Q1	Q3	N	CI	p-value
DISCERN 1	Hand surgeon	26.49	26.0	6.35	22.0	30.0	41	1.94	0.017
	Physiotherapist	22.06	22.0	4.32	19.0	25.0	35	1.43	
	Physician	23.94	24.5	3.73	21.8	27.0	16	1.83	
	Orthopedist	23.31	24.0	5.85	22.0	26.0	13	3.18	
	Occupational therapist	21.82	23.0	5.34	18.5	23.5	11	3.16	
DISCERN 2	Hand surgeon	24.73	24.0	5.93	20.0	29.0	41	1.81	0.002
	Physiotherapist	20.49	20.0	3.81	18.0	22.0	35	1.26	
	Physician	22.38	22.0	4.27	20.8	23.5	16	2.09	
	Orthopedist	21.69	22.0	4.57	21.0	24.0	13	2.48	
	Occupational therapist	19.45	19.0	4.41	17.5	20.0	11	2.61	
GQS 1	Hand surgeon	2.39	2.0	1.00	2.0	3.0	41	0.31	0.071
	Physiotherapist	1.80	2.0	0.72	1.0	2.0	35	0.24	
	Physician	2.06	2.0	0.57	2.0	2.0	16	0.28	
	Orthopedist	2.00	2.0	0.71	2.0	2.0	13	0.38	
	Occupational therapist	2.00	2.0	0.77	2.0	2.0	11	0.46	
GQS 2	Hand surgeon	2.37	2.0	0.99	2.0	3.0	41	0.30	0.011
	Physiotherapist	1.69	2.0	0.68	1.0	2.0	35	0.22	
	Physician	2.00	2.0	0.63	2.0	2.0	16	0.31	
	Orthopedist	1.92	2.0	0.64	2.0	2.0	13	0.35	
	Occupational therapist	1.64	1.0	0.92	1.0	2.0	11	0.55	

**Table 6:** Post-hoc p-values among the professionals.

		Hand surgeon	Physiotherapist	Physician	Orthopedist
DISCERN 1	Physiotherapist	0.002			
	Physician	0.226	0.076		
	Orthopedist	0.239	0.300	0.895	
	Occupational therapist	0.033	0.806	0.144	0.383
DISCERN 2	Physiotherapist	0.001			
	Physician	0.293	0.048		
	Orthopedist	0.201	0.155	0.930	
	Occupational therapist	0.005	0.254	0.011	0.054
GQS 2	Physiotherapist	0.002			
	Physician	0.236	0.109		
	Orthopedist	0.171	0.249	0.742	
	Occupational therapist	0.021	0.560	0.092	0.170

Let us take as an example the result for DISCERN 1, where the highest mean was for Hand Surgeon, with a mean of 26.49, which, based on the post-hoc p-values, is statistically different from the Physiotherapist, with a mean of 22.06 (p-value = 0.002), and from the Occupational Therapist, with a mean of 21.82 (p-value = 0.033).

In addition to these data, the type of publication was analyzed and compared for the scores of each evaluator, which is shown in Tables 7 and 8. We divided the publications into 3 types: photo, Reels (short video), and carousel (a post with more than 1 photo). In the data analysis, it was evidenced that there are statistically significant differences among the types of publications (p-values in Table 8).

**Table 7:** Comparison of Publication Type for DISCERN and GQS.

		Mean	Median	Standard Deviation	Q1	Q3	N	CI	p-value
DISCERN 1	Carousel	25.19	24.0	5.34	21.5	27.3	16	2.62	0.010
	Photo	22.07	22.0	4.46	19.0	25.0	45	1.30	
	Reels	25.23	25.0	6.03	22.0	29.0	57	1.56	
DISCERN 2	Carousel	22.44	21.5	3.69	19.0	25.0	16	1.81	0.014
	Photo	20.47	20.0	4.31	18.0	22.0	45	1.26	
	Reels	23.60	22.0	5.69	20.0	28.0	57	1.48	
GQS 1	Carousel	2.13	2.0	0.72	2.0	2.0	16	0.35	<0.001
	Photo	1.71	2.0	0.66	1.0	2.0	45	0.19	
	Reels	2.39	2.0	0.88	2.0	3.0	57	0.23	
GQS 2	Carousel	1.94	2.0	0.77	1.8	2.0	16	0.38	<0.001
	Photo	1.62	2.0	0.68	1.0	2.0	45	0.20	
	Reels	2.30	2.0	0.89	2.0	3.0	57	0.23	

**Table 8:** Post-hoc p-values among the publication types.

			Carousel	Photo
Trigger finger	DISCERN 1	Photo	0.062	
		Reels	0.718	0.004
	DISCERN 2	Photo	0.085	
		Reels	0.583	0.005
	GQS 1	Photo	0.051	
		Reels	0.274	<0.001
	GQS 2	Photo	0.141	
		Reels	0.124	<0.001

Analyzing Table 8, we found that in the comparisons performed the difference is observed between Photo and Reels, with photo-type publications always yielding the lowest mean and Reels-type publications the highest, as evidenced in the DISCERN 1 analysis with means of 22.07 and 25.23 (p-value = 0.004), respectively.

## DISCUSSION

It is a fact that social media and digital platforms make available a great deal of information related to medical content. However, most of this information lacks technical and scientific validation, which can lead to mistaken guidance for patients.

Data on trigger finger available on YouTube have already been analyzed in other previous studies, and it was demonstrated that they are of low quality, unreliable, and not very instructive.^16,17^ As Instagram posts related to these subjects have not yet been evaluated, this work set out to determine the quality of this information using two different objective scores, the modified DISCERN and the GQS. In addition, we sought to evaluate inter-observer reliability, considering the results indicated by two orthopedic physicians who are residents in hand surgery.

It is worth noting that the social network Instagram was selected because it is one of the most widely used social media platforms in Brazil today.

To carry out the study, we chose to create on the network a profile of a 50-year-old woman, corresponding to the sex and age most commonly affected by trigger finger^5,18^.

The results demonstrated high inter-rater reliability for both instruments used (DISCERN and GQS), reinforcing the robustness of the assessment applied to the analyzed posts. These data are consistent with data already existing in the literature, which evidence adequate inter-observer reliability for already-validated assessment methods, such as DISCERN.^15^

The positive, although weak, correlation between video duration and the quality measured by the scores suggests that longer content tends to present greater informational rigor, which may be related to the possibility of addressing the topic in greater depth. However, the magnitude of this correlation indicates that video length alone does not guarantee information quality.

The analysis between the number of views (low vs. high) and the quality of the posts showed no significant differences, suggesting that the popularity of a piece of content is not necessarily associated with its scientific quality. This finding is relevant, as it shows that engagement metrics, commonly valued on social media, should not be used as indicators of reliability for the lay or professional public.

The comparison among the different professionals revealed statistically significant differences, especially in the DISCERN and GQS scores. Posts by hand surgeons obtained the best results, presenting higher quality than those produced by physiotherapists and occupational therapists. This difference may be attributed to the greater technical and scientific mastery of hand surgeons over the topics addressed, as well as to academic training oriented toward producing more structured content aligned with scientific evidence.

On the other hand, the lower score observed among physiotherapists and occupational therapists should not be interpreted as an absence of relevance of these professionals in the rehabilitation process, but may indicate a need for greater training in the use of social media for disseminating evidence-based information.

These findings align with previous studies that point to heterogeneity in the quality of health information available on digital platforms.^14^ Moreover, they reinforce the importance of media education both for health professionals — in the sense of developing scientific communication skills adapted to the lay public — and for users, who need to be empowered to identify more reliable sources.

Other results demonstrated a statistically significant difference among the publication formats (carousel, photo, and Reels) for both evaluators and for both analysis instruments, DISCERN and GQS (p < 0.05).

Consistently, the Reels format presented the highest quality means, while the photo format presented the lowest values. This pattern was confirmed in the post-hoc analysis, which identified a significant difference specifically between photo and Reels in all comparisons (for example, DISCERN 1: 22.07 vs. 25.23; p = 0.004).

These findings suggest that the visual presentation format influences not only the reach of the posts — as already demonstrated in studies on digital engagement — but also the perceived and measurable quality of health information.

The Reels format allows greater exposure time, the use of verbal explanations, and the inclusion of dynamic visual elements, which can contribute to greater detailing of the educational content. In contrast, the photo format depends almost exclusively on the text written in the caption, which is often simplified, superficial, or reduced to promotional messages.

The overall DISCERN means were 24.02 for evaluator 1 and 22.25 for evaluator 2, with GQS scores of 2.09 and 1.99 for evaluators 1 and 2, respectively.

Olcar^16^ and Uzel et al^17^ concluded in their studies that YouTube videos about trigger finger generally present low quality and low reliability as a source of information for patients. The analysis of the most-viewed videos, by means of scores, demonstrated low means across all instruments, classifying most of the content as inadequate or of little use for health education.

Considering that DISCERN can reach up to 80 points and that the GQS score ranges from 1 to 5, these results demonstrate that most of the publications do not meet minimum quality criteria to adequately guide patients, especially with regard to the explanation of treatment options, risks, benefits, and grounding in scientific evidence.

From the standpoint of health education, these results are concerning, especially considering that patients use social media as a primary source of information and decision-making when seeking medical care. Excessive simplification of content may contribute to the creation of unrealistic expectations, inappropriate self-diagnosis, or adherence to practices not validated by the scientific literature.

Another relevant aspect is that, despite the growth in the use of social media by health professionals, the results indicate that not all content produced meets technical quality criteria — and that the chosen format can be decisive for this. This finding reinforces the importance of qualified professionals, including hand surgeons and orthopedic specialists, acting as content producers, applying principles of efficient scientific communication geared toward the digital environment.

## CONCLUSION

The results of this study indicate that information about trigger finger (stenosing tenosynovitis) published on the Instagram platform is of unsatisfactory quality. A predominance of content with low quality and reliability, limited scientific basis, and restricted clinical usefulness for the lay public was observed, a fact corroborated by the low scores achieved on the assessment instruments used.

Thus, this work demonstrates an important gap between the public's need for quality information and what is actually made available. This gap represents an opportunity for the work of specialists, the dissemination of evidence-based content, and the strategic use of the most effective formats for transmitting information.

## Data Availability

The data underlying the research text are contained in the manuscript.
